# Single-cell Profiling of Reprogrammed Human Neural Stem Cells Unveils High Similarity to Neural Progenitors in the Developing Central Nervous System

**DOI:** 10.1007/s12015-024-10698-3

**Published:** 2024-03-22

**Authors:** Angeliki Spathopoulou, Martina Podlesnic, Laura De Gaetano, Elena Marie Kirsch, Marcel Tisch, Francesca Finotello, Ludwig Aigner, Katharina Günther, Frank Edenhofer

**Affiliations:** 1 Department of Molecular Biology & CMBI, Genomics, Stem Cell & Regenerative Medicine Group, University of Innsbruck, Technikerstraße 25, 6020 Innsbruck, Austria; 2 Institute of Molecular Regenerative Medicine, Paracelsus Medical University, Salzburg, Austria; 3 Center for Stroke Research, Charité – Universitätsmedizin Berlin, Berlin, Germany; 4 Department of Experimental Neurology, Charité – Universitätsmedizin Berlin, Berlin, Germany; 5 Department of Molecular Biology, Digital Science Center (DiSC), University of Innsbruck, Innsbruck, Austria

**Keywords:** CNS development, Neural regeneration, Cellular benchmarking, Neural stem cells, Neuroepithelial cells, Reprogramming

## Abstract

**Background:**

Similar to induced pluripotent cells (iPSCs), induced neural stem cells (iNSCs) can be directly converted from human somatic cells such as dermal fibroblasts and peripheral blood monocytes. While previous studies have demonstrated the resemblance of iNSCs to neural stem cells derived from primary sources and embryonic stem cells, respectively, a comprehensive analysis of the correlation between iNSCs and their physiological counterparts remained to be investigated.

**Methods:**

Nowadays, single-cell sequencing technologies provide unique opportunities for in-depth cellular benchmarking of complex cell populations. Our study involves the comprehensive profiling of converted human iNSCs at a single-cell transcriptomic level, alongside conventional methods, like flow cytometry and immunofluorescence stainings.

**Results:**

Our results show that the iNSC conversion yields a homogeneous cell population expressing bona fide neural stem cell markers. Extracting transcriptomic signatures from published single cell transcriptomic atlas data and comparison to the iNSC transcriptome reveals resemblance to embryonic neuroepithelial cells of early neurodevelopmental stages observed in vivo at 5 weeks of development.

**Conclusion:**

Our data underscore the physiological relevance of directly converted iNSCs, making them a valuable in vitro system for modeling human central nervous system development and establishing translational applications in cell therapy and compound screening.

**Graphical Abstract:**

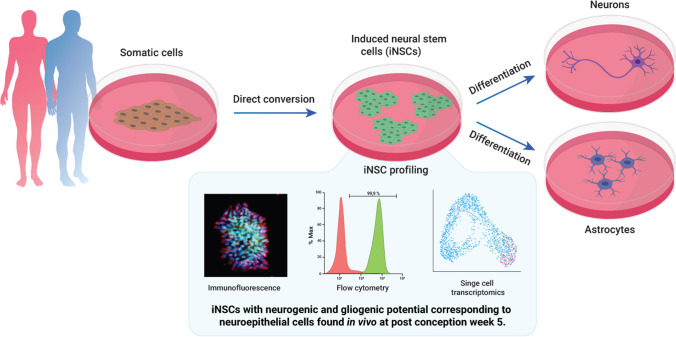

**Supplementary Information:**

The online version contains supplementary material available at 10.1007/s12015-024-10698-3.

## Introduction

Neural stem cells (NSCs) are multipotent, self-renewing stem cells that respond to various temporal and spatial cues during early embryonic development in order to generate the organism’s nervous system [1–3]. Postnatally, NSC populations decline but in many species, including humans, a rare population of adult NSCs is maintained in the neurogenic niches of the adult brain throughout life [4, 5]. Adult NSCs remain mostly inactive in a quiescent state and only become activated through extrinsic and intrinsic niche cues to give rise to neuroblasts, which then further differentiate into neurons [6]. Despite the identification of these cellular populations, many aspects around their mechanisms of activation and their role in the central nervous system (CNS) regeneration or neurodegeneration remain elusive. Addressing these open questions is challenging when relying on the currently existing model systems. The access to human post-mortem brain specimens is limited and these tissues are in a terminal, static condition. Therefore, modeling the complex, transient changes during NSC activation and differentiation is difficult. Thus, developing in vitro systems that mirror neurodevelopment, adult neurogenesis and brain regeneration is highly desired.

Reprogramming of somatic cells into patient-derived induced pluripotent stem cells (iPSCs) through the overexpression of the so-called Yamanaka OSKM factors opened unique opportunities for biomedical research [7, 8]. Currently, iPSCs and their differentiated progenies are routinely used for basic research, disease modeling and compound screenings. However, iPSCs are accompanied by some disadvantages, including the rejuvenation occurring during the reprogramming process, which erases epigenetic marks present in the donor’s genome [9]. Additionally, iPSCs harbor the risk of teratoma formation upon transplantation and require further downstream differentiation into the neural lineage with extrinsic patterning cues. Transdifferentiation methods, like the direct conversion into induced neurons (iNs) [10, 11], resolve some of the aforementioned drawbacks, such as the cellular rejuvenation [12]. However, iNs are post-mitotic cells and thus not self-renewable. Therefore, the transdifferentiation process needs to be repeated continuously from scratch, a process that is laborious and results in substantial biological variability of the transdifferentiation outcomes [13]. Moreover, post-mitotic iNs do not model the regenerative capacity of the somatic NSCs, which are physiologically present in the mammalian CNS. Conversion of somatic cells into induced NSCs (iNSCs) has the potential to overcome these limitations [14–19]. iNSCs are multipotent neural stem cells, self-renewing under clonal conditions and derived in vitro from juvenile or adult skin-derived fibroblasts. iNSCs are generated following a controlled short-term exposure of the cells to the OSKM factors, in combination with a chemically-defined neural induction medium (NIM) [14]. Despite the use of OSKM factors for the iNSC derivation, they do not accomplish a pluripotency stage and therefore could be a promising tool for clinical applications in an autologous manner [20]. In spite of the various successful applications of iNSCs in disease modeling, neurodevelopmental research, as well as cell sources for pre-clinical cell replacement therapies [21–27], their functional status, particularly when compared to their physiological NSC counterparts remains largely unknown. In this study, we aim to assess a precise transcriptomic profile of the in vitro derived human iNSCs and to identify genes that are shared with the physiologically present NSCs, as well as factors that discriminate them from their natural counterparts. To this aim, we performed single-cell transcriptomic analyses, accompanied by data from flow cytometry and immunofluorescence stainings. Moreover, we perform state-of-the-art analyses that compare the transcriptomic signatures of the iNSCs with published transcriptomic atlases, covering embryonic brain samples and multiple other human tissues [28–30]. Our study shows that iNSCs directly converted from human skin fibroblasts represent a highly homogeneous cellular population that expresses bona fide NSC markers and is transcriptomically similar to embryonic neuroepithelial cells of early neurodevelopment.

In conclusion, we provide a detailed benchmarking of the developmental and cellular identity of in vitro derived iNSCs and their in vivo counterparts. This feature has been missing from the published literature to date, but will be essential for the improved and safe use of iNSCs in basic research and translational applications.

## Materials and Methods

### Cell Culture

All adult human fibroblast (ADF) lines were derived from commercially available sources (ATCC). All cell lines were cultured at 37 °C, 5% CO_2_ conditions. ADFs were maintained with fibroblast medium, DMEM (Thermo Fisher Scientific), 15% FBS (Thermo Fisher Scientific) and 1 × non-essential amino acids (Sigma). The medium was changed every second day and the cells were passaged using Trypsin–EDTA (Sigma). ADFs were frozen in 90% FBS supplemented with 10% DMSO (Roth) and stored at -80 °C until further usage.

Induced neural stem cells (iNSCs) were cultured on GFR matrigel (Corning) or Geltrex (Thermo Fischer Scinetific) coated plates with neural induction medium (NIM), 1:1 mixture of DMEM/F12 (Thermo Fisher Scientific) to Neurobasal (Thermo Fisher Scientific), 1 × N2 (Thermo Fisher Scientific), 1 × B27 supplemented with Vitamin A (Thermo Fisher Scientific), 1% GlutaMAX (Thermo Fisher Scientific), 10 ng/ml hLIF (Thermo Fisher Scientific), 2 μM SB431542 (THP) and 3 μΜ CHIR99021 (Axon Medchem). The medium was changed every second day and the cells were passaged using Accutase (Sigma). iNSCs were frozen in KnockOut™ serum replacement (Thermo Fisher Scientific), supplemented with 10% DMSO and stored at -80 °C until further usage. The lines were regularly tested for mycoplasma contamination.

### Neural Stem Cell Derivation

iNSCs were converted from ADFs as previously described [14] with slight modifications. Briefly, ADFs were plated in a density of 1.55 × 10^4^ cells/cm^2^ and one day later transduced using the Cytotune 2.0 Sendai kit (Thermo Fisher Scientific) with a MOI of 3 for each viral preparation. After 24 h the viral medium was aspirated and the cells were supplemented with NIM medium and were maintained at 39 °C, 5% CO_2_ for 14 days. The medium was changed every other day. On day 7 of the conversion process the cells were harvested using Accutase (Sigma) and replated on a freshly coated GFR Matrigel 6well. From day 14 onwards the cells were returned at the typical culturing conditions of 37 °C, 5% CO2. iNSC colonies started to appear around day 17 and once they reached a sufficient size, they were manually isolated and further expanded.

### Neuronal Differentiation

iNSCs were differentiated following the protocol by Reinhardt et al*.* [31] with minor modifications. Briefly, iNSCs were plated on 15 μg/ml poly-L-ornithine (PLO, Sigma)/ 1µg/ml Laminin (Sigma) coated plates. For the first two weeks of the differentiation the cells were cultured with 1:1 mixture of DMEM/F12 to Neurobasal, 1 × GlutaMAX, 1 × N2, 1 × B27 supplemented with Vitamin A, 20 ng/mL BDNF (Miltenyi), 20ng/mL GDNF (Miltenyi), 300 ng/mL cAMP (THP), 200 μM ascorbic acid (Sigma) and 2 μM DAPT (THP). The culture medium was switched for weeks 3 and 4 of the differentiation to 1:1 mixture of DMEM/F12 to Neurobasal, 1 × GlutaMAX, 1 × N2, 1 × B27 supplemented with Vitamin A, 20 ng/mL BDNF (Miltenyi), 20 ng/mL GDNF (Miltenyi), 300 ng/mL cAMP (THP) and 200 μM ascorbic acid (Sigma). The medium was half changed every second day during the whole duration of the differentiation.

### Astrocyte Differentiation

iNSCs were differentiated into immature astrocytes following the previously described protocols from Appelt-Menzel et al*.* and Reinhardt et al. [31, 32]. Briefly, iNSCs were cultured on GFR Matrigel (Corning)-coated plates for two days in FGF/ EGF medium (1:1 mixture of DMEM/F12 (Thermo Fisher Scientific) to Neurobasal (Thermo Fisher Scientific), 2 mM L-Glutamine (Thermo Fisher Scientific), 1 × B27 supplemented with Vitamin A (Thermo Fisher Scientific), 1 × N2, 10 ng/mL bFGF (Thermo Fisher Scientific), 10 ng/mL EGF (Peprotech). From day 3 onwards culture medium was switched to astrocyte differentiation medium (DMEM/F12 (Thermo Fisher Scientific), 1 × GlutaMAX (Thermo Fisher Scientific), 1 × N2 (Thermo Fisher Scientific), 4% FBS (Thermo Fisher Scientific), 10 ng/mL CNTF (Thermo Fisher Scientific)) until day 16 of the differentiation. At confluency, the cells were split using Accutase (Sigma).

### Flow Cytometry

Cells were washed with cold 1 × PBS and then detached into a single cell suspension using Accutase (Sigma). The cell number was determined and at least 5 × 10^5^ cells were used per staining. In all following steps the cell preparations were protected from light. For each cellular marker the appropriate isotype control and an unstained sample were analyzed as well. After harvesting, the cells were stained with the viability dye eBioscience Fixable Viability Dye eFluor 780 (Thermo Fischer Scientific) in a 1:1000 dilution in cold 1 × PBS for 30 min at 4 °C. Subsequently, the cells were centrifuged at 300 × g for 3 min at 4 °C. The cell pellet was washed twice with cold 1 × PBS. For the cell surface marker staining each antibody was used in a 1:50 dilution in cold 1 × PBS and the staining was performed for 15 min at 4 °C. The cells were washed once with cold 1 × PBS and resuspended in 200 μl cold 1 × PBS. Finally, the cells were analyzed with the CytoFLEX flow cytometer (Beckman) and 3 × 10^5^ cells were recorded per sample. The analysis was performed with the Kaluza software (Beckman). The antibodies and isotype controls that were used are summarized in Table 1.

**Table 1:** List of flow cytometry antibodies

Antibody	Dilution	Manufacturer
Anti-human CD184 (CXCR4) PE/ Cyanine5	1:50	BioLegend
Anti-human/mouse/rat PSA-NCAM PE	1:50	Miltenyi
Anti-human CD133/1 PE, REAfinity™	1:50	Miltenyi
Isotype Control Antibody, mouse IgG1, PE/ Cyanine5	1:50	Invitrogen
Isotype Control Antibody, mouse IgM, PE	1:50	Miltenyi
Isotype Control Antibody, human IgG1, REAfinity™	1:50	Miltenyi

### Immunofluorescence Stainings and Microscopy

The cells were seeded on HNO_3_ treated and GFR matrigel (Corning®) or Geltrex (Thermo Fischer Scinetific) coated glass coverslips. Once at the right confluency the cells were fixed with 4% PFA (Sigma) for 15 min at room temperature (RT). The cells were washed three times with PBST, i.e., 1 × PBS supplemented with 0.2% Triton X-100 (Sigma), for 5 min each. Subsequently, the cells were incubated in blocking solution (PBST with 3% BSA (Applichem) for 1 h at RT. After the blocking step the primary antibody solution (primary antibody diluted in blocking buffer) was added and the cells were incubated overnight in the fridge. The next day the cells were washed three times with PBST for 5 min each and then the secondary antibody solution was added, i.e., secondary antibody diluted in PBST supplemented with 1.5% BSA. The cells were incubated for 2.5 h at RT. The secondary antibody solution was removed and the cells were washed three times in PBST, for 5 min each. Finally, the nuclei were counterstained with DAPI solution (Thermo Fisher Scientific) for 5 min at RT. The cells were washed twice with PBST and once with PBS and were mounted on a glass slide using Aqua-Poly/Mount (PolyScience) or Prolong Antifade (Invitrogen) mounting medium. The antibodies that were used are summarized in Table 2. Immunofluorescence imaging was performed using the Leica DMi8 microscope and image processing was done in Fiji [33].

**Table 2:** List of immunofluorescence antibodies

Antibody	Dilution	Manufacturer
Anti-NESTIN, mouse monoclonal IgG	1:500	R&D Systems
Anti-PAX6, rabbit polyclonal IgG	1:100	Biolegend
Anti-SOX1, goat polyclonal IgG	1:200	R&D Systems
Anti-MAP2, mouse monoclonal IgG	1:100	Sigma
Anti-TUBB3, rabbit monoclonal IgG	1:1000	Abcam
Anti-S100ß, rabbit monoclonal IgG	1:400	Merck
Anti-GFAP, guinea pig polyclonal IgG	1:500	Invitrogen
Donkey Anti-goat IgG Secondary antibody, Alexa Fluor 594	1:1000	Thermo Fisher Scientific
Donkey anti-mouse IgG Secondary antibody, Alexa Fluor 647	1:1000	Thermo Fisher Scientific
Donkey anti-rabbit IgG Secondary antibody, Alexa Fluor 568	1:1000	Thermo Fisher Scientific
Donkey anti-mouse IgG Secondary antibody, Alexa Fluor 594	1:1000	Thermo Fisher Scientific
Donkey anti-rabbit IgG Secondary antibody, Alexa Fluor 488	1:1000	Thermo Fisher Scientific
Donkey anti-goat IgG Secondary antibody, Alexa Fluor 488	1:1000	Thermo Fisher Scientific

## Bioinformatics Analysis

### Library Preparation

Cells were harvested in a single cell suspension using accutase and the cell number and viability were defined with a Neubauer chamber. The viability of the cell suspension was > 95%. The library was prepared with the Chromium Controller (10 × GENOMICS), using the Chromium Next GEM Single Cell 3’ kit, v3.1 (10 × GENOMICS), following the manufacturer’s guidelines.

### Data Processing

The 10 × Genomics Cell Ranger 6.1.1 tool (https://www.10xgenomics.com/) was used to pre-process the raw single-cell RNA sequencing data; reads were aligned to the GRCh38 human genome and quantified to construct a gene-by-cell expression matrix. To exclude low-quality cells, barcodes expressing < 200 and > 8,000 genes were excluded from further analysis using Seurat (v4.3) [34]. Moreover, to ensure a high viability, cells expressing > 15% mitochondrial genes were removed. 1,659 over 1,751 cells passed the quality thresholds and were included in the downstream analysis.

### Dimensionality Reduction, Cell Clustering and Differential Gene Expression Analysis

Downstream analysis was performed using Seurat (v4.3) [34]. Briefly, log-normalization, scaling, cell cycle phase assignment and regression, clustering and differentially expressed gene (DEG) analysis were implemented. In order to reduce background noise, the log-normalized data were reduced to the first 2,000 most highly variable genes. After scaling, principal component analysis (PCA) was performed and the first 10 dimensions were selected. A resolution of 0.2 was selected and the data were visualized with a uniform manifold approximation and projection (UMAP) for dimensionality reduction. Differentially expressed genes of the resulting clusters were determined by applying the function FindVariableGenes, using an adjusted p-value < 0.05 and a log2FC > 1. Gene enrichment analysis over Gene Onthology (GO) terms and KEGG pathways of DEGs were generated by was performed using ShinyGo (v0.77) [24].

### Cell Annotation

In order to annotate the iNSC dataset in an unbiased manner, SingleR (v1.8.1) was used employing several publicly available studies as reference for the cell type and developmental age annotations [35]. In detail, the transcriptomic atlases used as references were published from Mabbott et al*.* [28], Eze et al*.* [29] and Zeng et al*.* [30]. Data were transformed into the single-cell experiment format by the package SingleCellExperiment (v1.18.0) and each single-cell of the iNSC dataset was compared to every existing annotation of the reference atlases [36]. Subsequent visualization was performed using the packages pheatmap (v1.0.12) and dittoSeq (v1.6.0) [37, 38].

### Integration of the iNSC scRNA Sequencing Data with Several Reference Atlases

Integrating the iNSC and fibroblast single-cell RNA sequencing data was performed using Seurat by reciprocal PCA. Both datasets were reduced to their common genes. The strength of the alignment, reflected by the parameter k.anchor, was set to 3 and the first 30 principal components were considered for the clustering. The integration of the iNSC data and the human embryonic brain single-cell data of Eze et al*.* [29] was performed using the bioinformatics tool scArches (v0.5.1) with the single-cell variational inference (scVI) in Python, according to the default settings [39, 40]. Before integration, the Seurat objects were transformed to anndata objects using the packages SeuratData (v0.2.2) and SeuratDisk (v0.0.0.9020) [41, 42].

### Single-Cell Trajectory Analysis

In order to perform single-cell trajectory analysis, the pipeline of the bioinformatics tool monocle3 (v1.3.1) was used on the annotated iNSC dataset [43–45]. The Seurat object of the annotated iNSC dataset was converted into the cell_data_set class. The root cells were defined as the cells belonging to the iNSC cluster.

## Results

### In vitro Converted iNSCs Uniformly Express Bona Fide NSC Markers

**Fig. 1:**
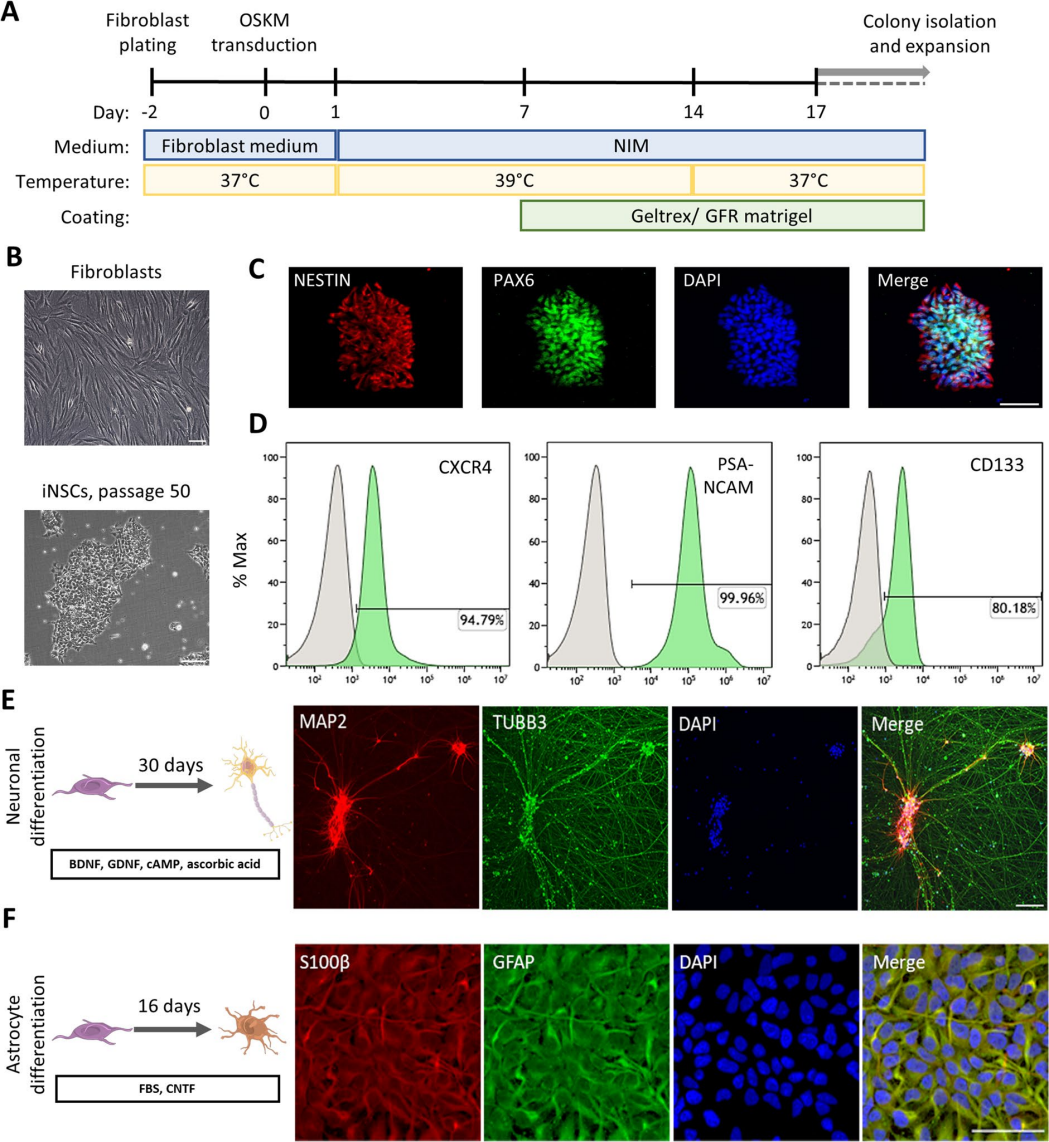
Induced neural stem cells (iNSCs) directly converted from human fibroblasts express bona fide NSC markers and maintain their neurogenic and gliogenic properties. **A**. Schematic representation of iNSC derivation. **B.** Phase contrast images of the parental dermal fibroblasts and the iNSCs derived thereof in culture. Scalebar: 200 μm. **C.** Immunofluorescence staining of bona fide NSC markers, NESTIN depicted in red, PAX6 in green, and nuclei in blue. Scalebar: 50 μm. **D**. Flow cytometric analysis of NSC markers, CXCR4, PSA-NCAM and CD133. The percentage of each positive marker is indicated in each individual plot. The grey peak represents the appropriate isotype control and the green each staining. x axis: PE-Area fluorescence in logarithmic scale. **E**. Immunofluorescence staining of neurons derived from iNSCs after 30 days of neuronal differentiation, MAP2 depicted in red, TUBB3 in green, and nuclei in blue. Scalebar: 100 μm. **F**. Immunofluorescence staining of immature astrocytes derived from iNSCs after 16 days of astrocytic differentiation, S100β depicted in red, GFAP in green, and nuclei in blue. Scalebar: 50 μm

To generate iNSCs we employed a controlled short-term exposure of somatic cells to the OSKM factors, which are delivered by Sendai virus (SeV) constructs in combination with a chemically-defined neural induction medium (Fig. 1A) [14]. Deactivation of the extrinsic OSKM factors was achieved by exposure of the converting cells to 39 °C for 14 days due to the temperature-sensitivity of SeV. The virus-associated RNA was not detectable after qPCR (Supp. Figure 1A–B). During the conversion, the cells transition from the elongated fibroblast morphology to a typical NSC-like morphology. This includes the formation of compact colonies that can be maintained in vitro for multiple passages (Fig. 1B) and express typical NSC markers, like NESTIN and Paired Box 6 (PAX6), when imaged after immunofluorescence stainings (Fig. 1C). Flow cytometry analysis of NSC-related surface markers demonstrated that the cells uniformly express C-X-C motif chemokine receptor 4 (CXCR4), an important protein for NSC proliferation and migration [46]. Almost 100% of the cells express polysialylated-neural cell adhesion molecule (PSA-NCAM), a marker known to be highly enriched in NSCs and immature neurons [47, 48]. Additionally, 80% of the cells express CD133, also known as Prominin-1 (PROM1), a glycoprotein typically expressed in NSC populations among other stem cell types [49] (Fig. 1D). Employing established in vitro differentiation paradigms, we show that converted NSCs maintain their neurogenic and gliogenic capacities, as judged by representative immunocytochemistry stainings with neuronal markers, Microtubule Associated Protein 2 (MAP2) and Tubulin Beta 3 Class III (TUBB3) (Fig. 1E), as well as astrocytic markers, like S100 Calcium Binding Protein B (S100β) and Glial Fibrillary Acidic Protein (GFAP) (Fig. 1F).

### Single-cell Transcriptomic Analysis Identifies a Homogenous iNSC Population

**Fig. 2:**
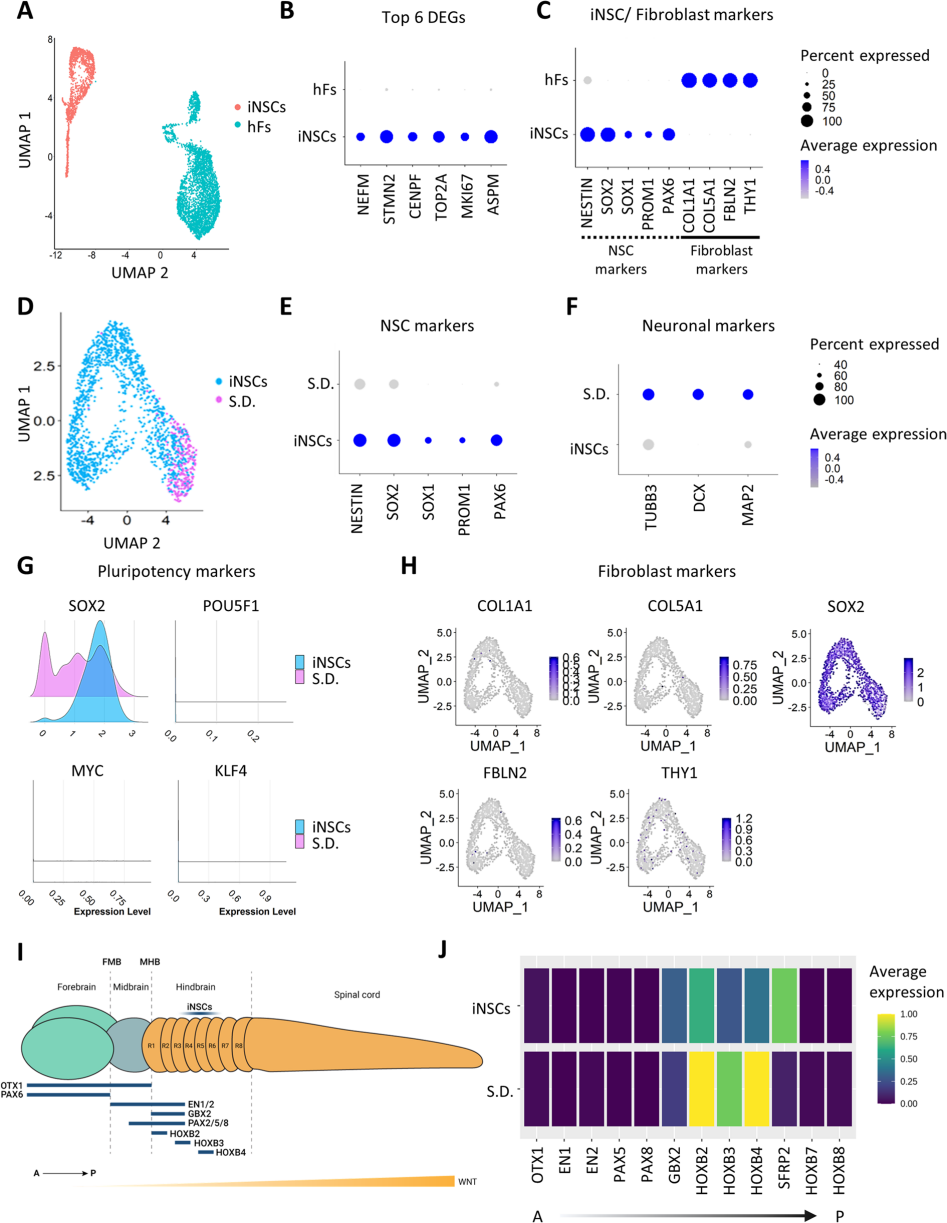
Transcriptomic profiling of iNSCs confirms that the generated cells are multipotent, express bona fide NSC markers and do not express markers of the parental fibroblasts. **A**. UMAP plot generated after the integration of iNSC and fibroblast single-cell RNA sequencing (scRNA-seq) data. Clustering based on their cellular identity, i.e., the originating fibroblasts or the derived iNSCs. **B**. Dotplot of the top 6 DEGs after the comparison of the iNSC versus fibroblast scRNA-seq data, showing an enrichment of NSC related genes, like the neurofilament component *NEFM*, the cytoskeleton gene *STMN2* and proliferation markers, like *TOP2A*, *CENPF*, *mKI67* and *ASPM* that are not present in the parental cell type. **C**. Dotplot of representative fibroblast and NSC markers generated from the integrated fibroblast/ iNSC data, depicting the clear distinction of the transcriptomic profiles of the originating and derived cell types. **D**. UMAP plot generated from iNSC scRNA-seq data based on the cellular identity. **E**. Dotplot of representative bona fide NSC markers, *NESTIN*, *SOX2*, *SOX1*, *PROM1*, and *PAX6*. **F**. Dotplot of representative neuronal markers, *TUBB3*, *DCX* and *MAP2*, showing expression of neuronal markers only at the spontaneously differentiating (s.d.) cellular cluster. The percentage of cells that are expressing each marker is indicated by the size of the dot, whereas the average expression is indicated with the color gradient. **G**. Ridge plots of pluripotency markers, *SOX2*, *POU5F1* (*OCT3/4*), *KLF4* and *cMYC*, indicating the sole expression of the multipotency marker *SOX2* and the absence of expression of the other three. **H**. Feature plots of representative fibroblast markers, *COL1A1*, *COL5A1*, *FBLN2* and *THY1*, showing absence of expression indicating the successful conversion of the iNSCs from fibroblasts. *SOX2* is depicted as a representative positive control. **I**. Schematic representation of the anterior/ posterior axis of the human neural tube. **J**. Heatmap of representative markers of the anterior/ posterior axis of the human neural tube, *OTX1*, *EN1*, *EN2*, *PAX5*, *PAX8*, *GBX2*, *HOXB2*, *HOXB3*, *HOXB4*, *SFRP2*, *HOXB7*, *HOXB8*. All plots are generated from the non-regressed scRNA-seq data. Abbreviations: hFs, human fibroblasts; S.D., spontaneously differentiating cells; DEGs, differentially expressed genes; FMB, forebrain/ midbrain boundary; MHB, midbrain/ hindbrain boundary; R, rhombomere; A, anterior; P, posterior

To confirm the successful and complete derivation of iNSCs from the parental fibroblasts, we first performed single-cell RNA sequencing (scRNA-seq) on both separate cellular populations. After integration of the two datasets and visualization on a uniform manifold approximation and projection plot (UMAP), it is apparent that the two different cell types cluster distinctively without any overlap of cellular identity (Fig. 2A). Unbiased comparison of their transcriptomic profiles revealed that the top differentially expressed genes (DEGs) of the iNSC cluster are NSC-related genes, like the neurofilament component *Neurofilament Medium Chain (NEFM)*, the cytoskeleton gene *Stathmin 2* (*STMN2*) and proliferation markers, like *DNA Topoisomerase II Alpha* (*TOP2A*), *Centromere Protein F* (*CENPF*), *Marker of Proliferation Ki-67* (*MKI67*) and *Assembly Factor for Spindle Microtubules* (*ASPM*) (Fig. 2B). Further investigation of the bona fide NSC markers, including *NESTIN*, *SOX2*, *SOX1*, *PROM1* and *PAX6* and fibroblast markers, *Collagen Type I Alpha 1 Chain* (*COL1A1)*, *Collagen Type V Alpha 1 Chain (COL5A1)*, *Fibulin 2* (*FBLN2)* and *Thy-1 Cell Surface Antigen* (*THY1)*, showed cell-type specific expression patterns (Fig. 2C), indicating a complete conversion resulting in a cellular population that exclusively expresses NSC markers.

Next, we asked whether iNSCs represent a homogeneous cell population rather than a mixture of committed neural precursors. To this end, we performed scRNA-seq and downstream analysis of 1,659 cells that successfully passed the quality control. Visualization of the data on a UMAP plot reveals four cellular clusters, which are separated based on the different cell cycle phases (Supp. Figure 2A, B, Supp. Figure 3). Indeed, regression of the cell-cycle related genes using the Seurat vignette “Cell-cycle Scoring and Regression” [50] resulted in a homogenous clustering on the UMAP plot (Supp. Figure 2C), a fact that is also reflected in the GO term analysis of the DEGs (Supp. Figure 4). Annotation of the cell-cycle phase of the cells revealed a uniform clustering, confirming that the cell-cycle gene regression was successful (Supp. Figure 2D). Further analysis of the transcriptomic profiles of the cellular clusters revealed an enrichment of early neuronal markers in a subpopulation of cluster 1 (Supp. Figure 2E), which was also reflected on the GO terms of this cluster (Supp. Figure 3B). Analysis of the transcriptomic identities of these clusters revealed two different cellular populations, iNSCs and spontaneously differentiated (S.D.) cells (Fig. 2D), with 83.66% of the total population being assigned to iNSCs and 16.34% being spontaneously differentiated cells. Investigation of bona fide human NSC markers revealed that the cells belonging to the iNSC cluster uniformly express a panel of NSC markers, including *NESTIN* (*NES*), *SRY* (*Sex* *Determining* *Region Y*)-*Box 2* (*SOX2*), *SOX1*, *PROM1*, and *PAX6* (Fig. 2E), many of which were also shown to be expressed at a protein level, as determined by ICC and flow cytometry, respectively (Fig. 1). Notably, the spontaneously differentiated cells do not exhibit any expression of NSC markers (Fig. 2E). Instead, the cells of this cluster express early neuronal markers, like *TUBB3*, *doublecortin* (*DCX*) and *MAP2*, further confirming the occurrence of a minor side population that spontaneously differentiates toward an early neuronal cell fate (Fig. 2F). We did not detect expression of pluripotency markers, including *POU5F1* (*OCT4*), *MYC* and *KLF4*, indicating that the cells are not in a pluripotent state. However, all iNSCs do express *SOX2*, a neural multipotency marker, which is essential for the NSC maintenance and proliferation (Fig. 2G) [51]. In addition, in order to assess the conversion success, we investigated the expression of markers related to the parental fibroblasts. The derived iNSCs do not maintain any expression remnants of fibroblast markers, e.g., *COL1A1*, *COL5A1*, *FBLN2* and *THY1*, indicating a complete conversion distinct from the original cell type (Fig. 2H).

At the beginning of the mammalian development, NSCs are important for the generation of the organism’s nervous system, a task fulfilled with a great complexity in spatial and temporal coordination. In humans, primary neurulation starts in developmental week 3 (D.W. 3) with the neural plate formation, invagination, and the closure of the neural tube, which later gives rise to the brain and spinal cord [52, 53]. At D.W. 5 the neural tube forms the three parts of the developing brain, forebrain, midbrain and hindbrain (Fig. 2I) [54]. In order to explore a putative spatial identity of the iNSCs, we analyzed the expression patterns of selected markers of the anterior/ posterior axis of the developing neural tube. Starting from the anterior forebrain and midbrain, iNSCs do not show expression of forebrain markers such as *Orthodenticle Homeobox 1* (*OTX1*) or midbrain markers, like *Engrailed Homeobox 1* (*EN1*), *Engrailed Homeobox 2 (EN2*), *PAX5* and *PAX8*. Moving more towards the posterior part of the neural tube, iNSCs were detected to express the hindbrain markers *Gastrulation Brain Homeobox 2* (*GBX2*), *Homeobox Protein A2* (*HOXA2*), *Homeobox Protein B2* (*HOXB2*), *Homeobox Protein B3* (*HOXB3*) and *Homeobox Protein B4* (*HOXB4*) (Fig. 2J). However, they do not express posterior hindbrain or spinal cord markers, like *Homeobox Protein B7* and *B8* (*HOXB7*, *HOXB8*), suggesting a spatial location of iNSCs at the anterior and middle parts of the hindbrain, at rhombomeres R2-R6 (Fig. 2I).

Taken together, our single-cell transcriptomic analyses reveal that the directly converted iNSCs consitute a homogenous stem cell population expressing established NSC markers and do not represent a heterogenous mixture of committed neural progenitors. Additionally, the iNSCs do not express any pluripotency-associated genes, but are multipotent as their physiological counterparts and are transcriptomically distinct from their parental fibroblasts. Finally, the cells express hindbrain-associated markers, further supporting the relevance of the in vitro population with in vivo developmental counterparts.

### Pseudotime Trajectory Analysis Reveals Dynamics of Neurogenic Instructor Genes

**Fig. 3:**
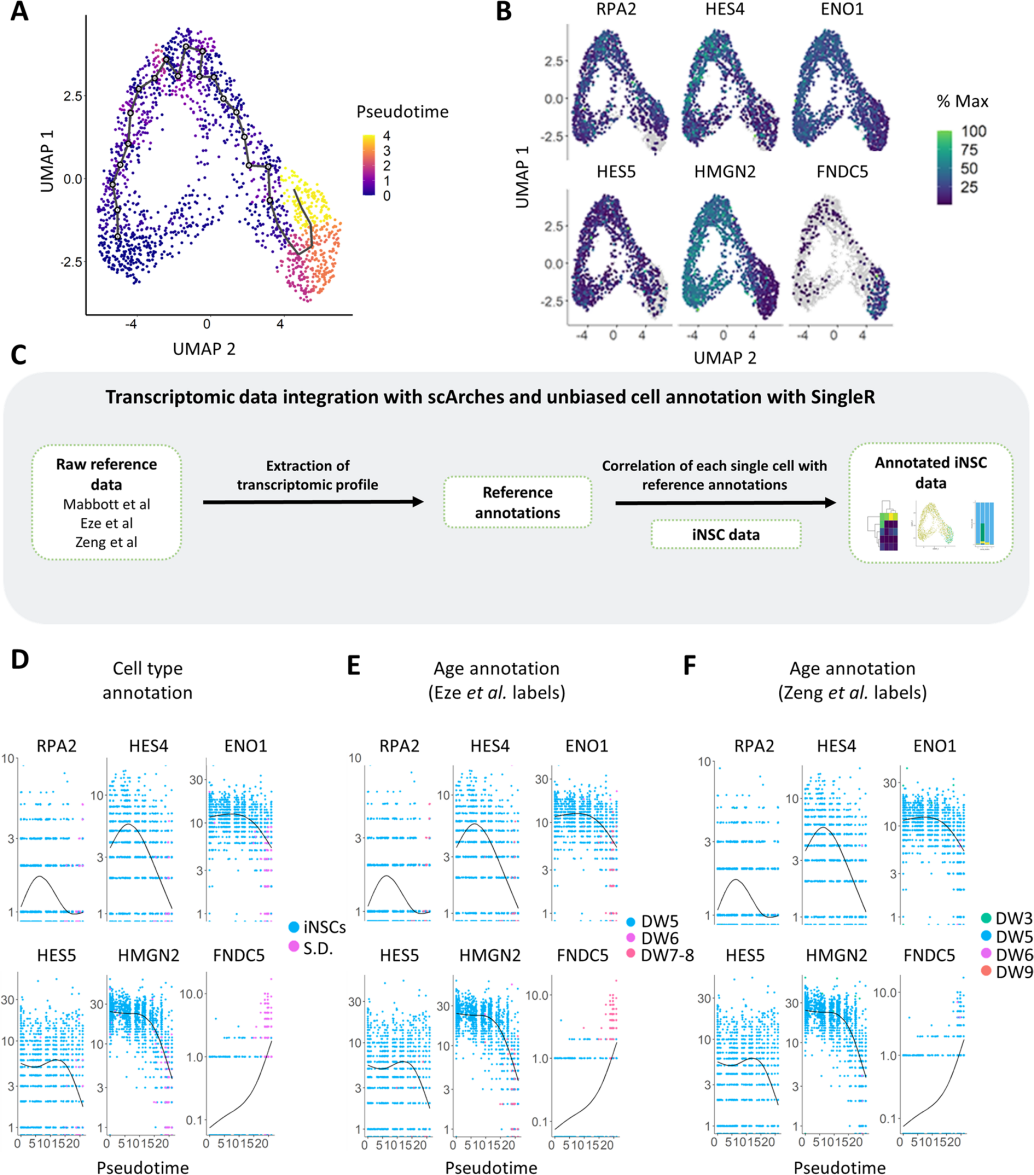
Trajectory analysis reveals a highly homogeneous NSC population and points out genes that are significant for the neuroepithelial/neuronal cell fate. **A**. UMAP plot generated from iNSC scRNA-seq data based on pseudotime trajectory analysis **B**. Top 6 DEGs that play an important role in the pseudotime trajectory. **C**. Schematic of the employed bioinformatics workflow, using the in silico tool SingleR for extracting transcriptomic signatures from the reference datasets and annotation of our iNSC transcriptomic data. **D**–**F.** Top 6 DEGs that play an important role in the pseudotime trajectory, annotated by cell type annotations (**D**), and by the age range of the Eze et al*.* reference dataset [13] (**E**) and the age of the Zeng et al*.* reference dataset (**F**) [30]. Abbreviations: S.D., spontaneously differentiating cells; D.W., developmental week

Next, we focused on the gene networks that induce and maintain iNSC identity. Trajectory inference methods can offer valuable insights into the gene dynamics that govern a biological process [45]. In this respect, we employed the Monocle3 in silico pipeline in order to investigate the genes that play an important role in the maintenance of the iNSC identity or drive the commitment towards a neuronal fate [45]. This analysis revealed that the pseudotime trajectory is initiated from the homogeneous iNSC cluster and progresses towards the differentiated neuronal subcluster (Fig. 3A). DEG analysis of the genes that play a crucial role in the pseudotime trajectory revealed several genes that are important for NSC proliferation and maintenance of stemness and one gene that directs the spontaneous differentiation of a small fraction of cells towards a neuronal commitment. *High Mobility Group Nucleosomal Binding Domain 2* (*HMGN2*), *Hes Family BHLH Transcription Factor 4* (*HES4*) and *Hes Family BHLH Transcription Factor 5* (*HES5*) [55–57], genes that are known to be important neuroepithelial markers in early mouse embryonic development [58], were stably expressed in the iNSC population and were diminished in the differentiated cells (Fig. 3B). Moreover, genes involved in DNA replication, like the *Replication Protein A2* (*RPA2*) [59], were among the identified DEGs, confirming the high proliferation status of the NSC population. *RPA2* is highly expressed in the NSC cluster and downregulated in the differentiating neuronal cluster (Fig. 3B), indicating the transition towards a post-mitotic cell fate. Conversely, *Fibronectin type III domain-containing protein 5* (*FNDC5*) was the only gene identified among the top 6 DEGs that plays an important role in the iNSC neuronal differentiation (Fig. 3B). Finally, *Enolase 1* (*ENO1*) was identified among the DEGs, a gene that encodes a protein involved in the glycolytic cycle (Fig. 3B). *ENO1* is enriched in NSCs and downregulated in the neuronal subcluster (Fig. 3B), an expected observation since neurons energetically rely on oxidative phosphorylation instead of glycolysis [60]. Further analysis of the gene dynamics between the two cellular populations showed that different genes play an important role in different stages. More specifically, investigation of the dynamics of gene expression in the two different cell types showed that most of the genes follow the same trajectory, exhibiting a stable expression in the iNSC cluster, with the exception of *FNDC5*, which is enriched at the spontaneously differentiated subfraction of cells (Fig. 3D) [61, 62].

Recent progress in NGS technologies allows the access to large, publicly available transcriptomic datasets from various human tissue-derived and embryonic specimens [63–65]. Such transcriptomic atlases can be employed as tools for benchmarking in vitro derived cellular populations, such as the iNSCs, with in vivo counterparts. In this regard, we employed the SingleR bioinformatics tool [35] in order to extract reference labels/signatures from three different transcriptomic atlases, generated by sequencing various human primary tissues [28] or human embryonic samples of various developmental stages [29, 30, 66]. Briefly, the dataset published from Mabbott et al*.* comprises an expression atlas consisting of microarray data from various primary tissues, including embryonic stem cells (ESCs), iPSCs, neuroepithelial cells and neurons [28]. The single cell transcriptomic atlas from Eze et al*.* is generated from embryonic whole brain specimens, ranging from D.W. 4 to 8 [29]. Lastly, the atlas published from Zeng et al*.* is comprised from scRNA-seq data from embryonic brain samples, spanning from D.W. 3–12 [30]. As shown in the pipeline schematic depicted in Fig. 3C, we used the reference signatures to annotate our iNSC transcriptomic data using the cell type or developmental age annotations before proceeding with the analysis and visualization of the annotated iNSC data.

**Fig. 4:**
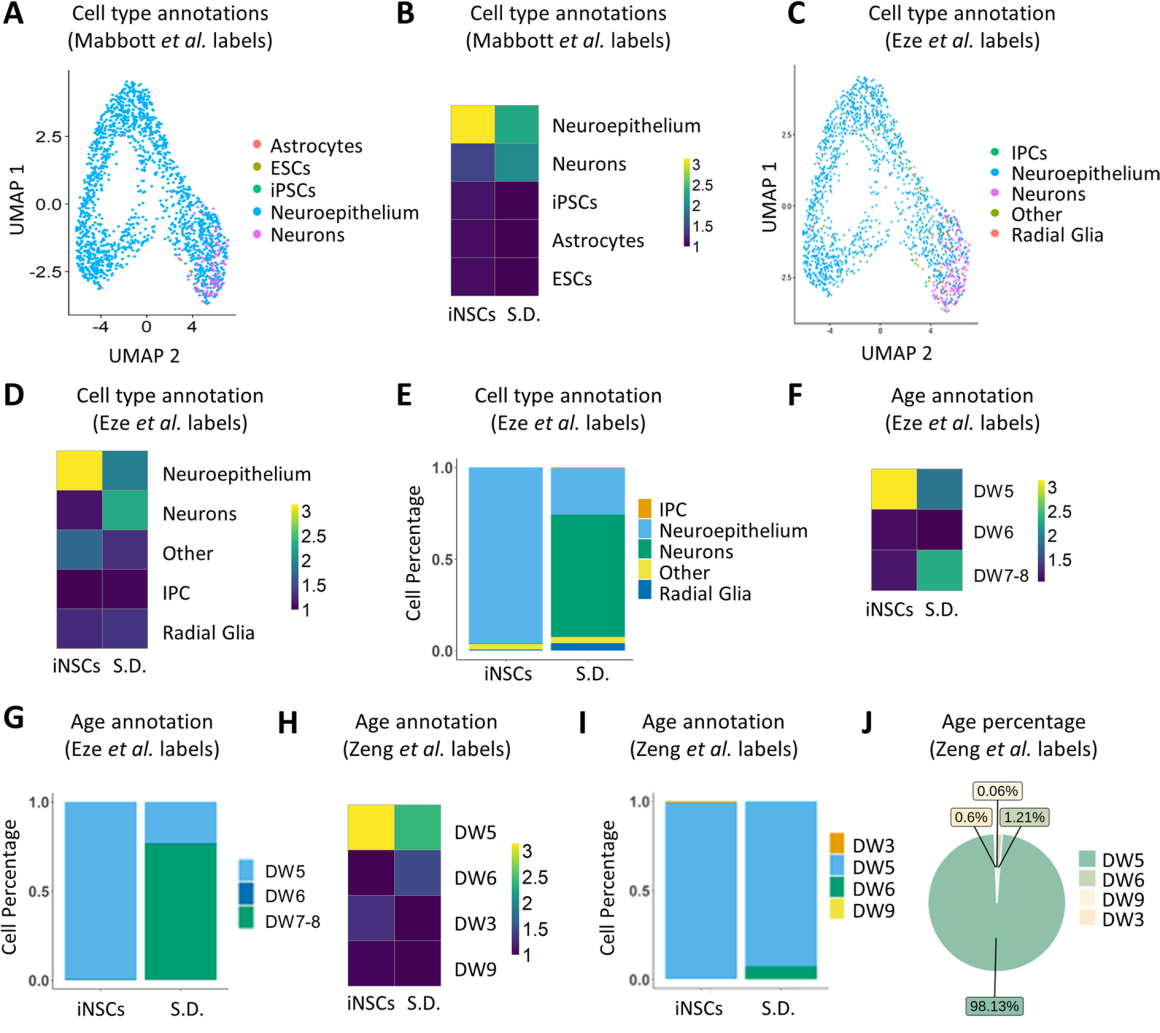
Comparative transcriptomic analysis of iNSCs and published transcriptomic datasets suggests a transcriptomic similarity of the converted iNSCs with human in vivo embryonic neuroepithelial cells during early neurodevelopment. **A**. UMAP plot generated from iNSC scRNA-seq data annotated with reference cell type labels. The reference cell type labels were extracted by the publication of Mabbot et al. [12] with the SingleR in silico tool [19] and then annotated onto the iNSC UMAP plot. **B**. Heatmap of the SingleR predicted cell type annotations from the iNSC UMAP clusters based on the Mabbott et al*.* labels. **C**. UMAP plot generated from iNSC scRNA-seq data annotated by reference cell type labels extracted with SingleR from the embryonic brain atlas publication of Eze et al. [13]. **D**. Heatmap exhibiting the cell composition of each Seurat cluster after the Eze et al*.* SingleR cell type annotation. **E**. Barplot exhibiting the cell composition of each Seurat cluster after the Eze et al. SingleR cell type annotation. **F**. Heatmap exhibiting the developmental age of each Seurat cluster after the Eze et al*.* SingleR cell type annotation. **G**. Barpot exhibiting the developmental age of each Seurat cluster after the Eze et al*.* SingleR age annotation. **H**. Heatmap exhibiting the developmental age of each Seurat cluster after the Zeng et al*.* SingleR age annotation [30]. **I**. Barpot exhibiting the developmental age of each Seurat cluster after the Zeng et al*.* SingleR age annotation. **J**. Pie chart exhibiting the fraction (%) of cells that belong to each developmental age based on the Zeng et al*.* SingleR age annotation. Abbreviations: S.D., spontaneously differentiating cells; D.W., developmental week

Investigation of the gene dynamics in correlation to the in vivo developmental age annotations extracted from 2 separate published embryonic atlases, led to the observation that the NSC-related genes are expressed in the early D.W. 5 cellular cluster, while *FNDC5* plays a role in the later developmental stages, which correlates with the physiological beginning of embryonic neurogenesis (Fig. 4D-F) [29, 30, 67]. Taken together, pseudotime trajectory analysis revealed genes that play an important role in induction and maintenance of iNSC stemness and further corroborated the commitment of a small subfraction of cells to a neuronal state.

### iNSCs are Transcriptomically Similar to Neuroepithelial Embryonic Cells from Early Neurodevelopmental Stages

Next, we investigated to which extent the in vitro derived and stabilized iNSCs do correlate to an equivalent found during in vivo human development. During neurulation, the neuroepithelium (NE) undergoes a particular transformation as it gives rise to neurons through a multi-step process involving radial glial cells (RG) and intermediate progenitor cells (IPC). RG cells serve as neural progenitors, providing structural support and guiding neuronal migration, while IPCs, derived from RGs, undergo further differentiation to generate a diverse population of neurons, contributing to the formation of the developing nervous system. Here, we asked to which extent the directly converted iNSCs mirror one of those physiological stages.

The analysis of the cell type annotations from both the Mabbott et al*.*, as well as the Eze et al*.* reference atlases uniformly attributes the iNSC cluster to a neuroepithelial cell type (Fig. 4A–E), with 93% of the cells being classified as neuroepithelial cells based on the Mabbott dataset (Supp. Figure 5A) and 84% based on the Eze dataset (Supp. Figure 5B). As anticipated, the small subset of the spontaneously differentiating cells (Fig. 2D) is indeed labeled as neuronal cells (Fig. 4A, C, Supp. Figure 5).

When comparing the iNSC transcriptomic profiles to the developmental stages found during embryonic development (Eze et al*.*), the iNSCs are annotated as cells correlated with embryonic samples of D.W. 5, while the neuronal subpopulation is correlated with later developmental stages of D.W. 7–8 (Fig. 4F, G). Comparing the iNSCs to the atlas published by Zeng et al*.,* which offers even greater resolution of the age of the specimens (3–12 D.W.), iNSCs are consistently transcriptomically similar to cells of D.W. 5 (Fig. 4H, I). The neuronal subcluster is as well annotated as D.W. 5, with a small fraction, 1,21%, being annotated as D.W. 6 (Fig. 4l).

In conclusion, the comparison of the in vitro derived iNSCs with embryonic transcriptomic reference atlases indicate that iNSCs are similar to human embryonic neuroepithelial cells present at developmental stage of D.W. 5.

## Discussion

Human iNSCs directly converted from somatic cells hold great promise for neurodevelopmental studies and a source for cell replacement therapies. Further progress in iNSC research critically depends on their comprehensive transcriptomic profiling and comparison to physiologic counterparts. Recently, embryonic scRNA-seq studies shed light into human brain development, revealing a great cellular complexity that was previously unknown [29, 30]. Despite the immense importance of these transcriptomic atlases, it remains crucial to establish in vitro platforms, as readily accessible models for studying human brain development and disease. Even though iPSCs are widely used to this end, they lack the physiological relevance to a developmental counterpart. Our study demonstrates that iNSCs represent an appropriate in vitro proxy for this purpose. By transcriptomic analyses at single cell level, we show that iNSCs represent a highly homogeneous population that uniformly expresses bona fide NSC markers. Furthermore, cellular benchmarking indicates that iNSCs are a multipotent, self-renewing stem cell population that in contrast to iPSCs does not exhibit pluripotency marker expression. Additional transcriptomic profiling revealed that the NSC conversion process is complete and successful, leading to a pure NSC population that is distinct from the original fibroblast cell type. Finally, the cells maintain their neurogenic and gliogenic capacities, differentiating into neurons and astrocytes in vitro. Transcriptomic profiling of several anterior/ posterior markers of the developing human neural tube revealed an expression of anterior and middle hindbrain markers, mainly expressed at rhombomeres R2-R6. This observation is in accordance with the patterning factors used for the iNSC maintenance, including factors for WNT activation, morphogens that are known to cause caudalization of the neural tube [68, 69].

Pseudotime trajectory analysis further elucidated the homogeneity of the main highly proliferative iNSC population and outlined the commitment of a small fraction of cells towards a small subpopulation of early differentiating neurons, rendering iNSCs a valuable system for modeling early neurogenesis. Several genes were identified as important factors for the maintenance of iNSC stemness, e.g., *HES4* and *HES5*, genes that are known to be important for the generation of the neuroepithelium during the early mouse development, but their role is not yet understood in humans [55, 56]. Conversely, *FNDC5* was identified as a driver towards the neuronal commitment, an important factor in neuronal differentiation in mice and human embryonic tissues [61, 62]. However, the exact mechanism of *FNDC5* biological function in human brain development is not fully elucidated.

Finally, we employed state-of-the-art in silico pipelines in order to extract transcriptomic signatures of different cell types present in the in vivo developing embryonic human brain. Subsequently, we compared these signatures to the in vitro derived NSCs and investigated whether these cells have transcriptomically similar cellular and developmental identities to the embryonic counterparts. Intriguingly, iNSCs are indeed transcriptomically similar to embryonic neuroepithelial cells of D.W. 5, with the subcluster of spontaneously differentiating cells being annotated as neuronal cells of either D.W. 4–8 (Eze et al*.* atlas) [29], or D.W. 5–6, based on the Zeng et al*.* reference dataset [30], emphasizing the potential of the model for the investigation of early neurogenesis.

## Conclusions

In summary, our data demonstrates that iNSCs represent a monoclonally expandable multipotent population of stem cells reflecting developmental age of 5 weeks of embryonic development. They represent a suitable, potentially autologous cellular population for studying early events of human brain development, as well as modeling pathoneurological phenotypes.

### Supplementary Information

Below is the link to the electronic supplementary material.

Supplementary file1 Supplementary Fig. 1 The derived iNSCs do not maintain Sendai-OSKM expression. **A**. qPCR data showing that the iNSCs do not maintain any expression of the Sendai-associated OSKM genes. Error bars represent the mean ±S.E.M., n =3 technical replicates per gene. The horizontal line represents the expression threshold. **B**. Agarose gels showing no expression of the Sendai-associated OSKM genes. Clones that express OSKM were used as positive controls.

High resolution image (TIF 764 kb)

Supplementary Fig. 2 UMAPs and heatmaps of the top 10 DEGs per cluster of the iNSC transcriptomic data. UMAP plots generated from iNSC scRNA-seq data clustered based on Seurat clustering (**A**) or their respective cell cycle phase (**B**). UMAP plots generated from iNSC scRNA-seq data after gene cell cycle regression. Clustering based on Seurat (**C**) or cell cycle phases (**D**), indicating a transcriptomically homogeneous NSC population after the cell cycle regression. Heatmap depicting the top 10 DEGs per cluster before (**E**) and after cell cycle gene regression (**F**).

High resolution image (TIF 10.8 mb)

Supplementary Fig. 3 GO terms per cluster of the iNSC transcriptomic data. Plots depicting the GO terms/ biological processes from cluster 0 (**A**), 1 (**B**), 2 (**C**) and 3 (**D**) as depicted in the UMAP plot of Supp. Fig. 2A. All plots are generated from the non-regressed scRNA-seq data. The plots are generated with the online bioinformatic tool ShinyGO 0.77.

High resolution image (TIF 2.06 mb)

Supplementary Fig. 4 GO terms per cluster of the cell cycle-regressed iNSC transcriptomic data. Plots depicting the GO terms/ biological processes from cluster 0 (**A**), 1 (**B**), and 2 (**C**) as depicted in the UMAP plot of Supp. Fig. 2C. All plots are generated from the cell cycle-regressed scRNA-seq data. The plots are generated with the online bioinformatic tool ShinyGO 0.77.

High resolution image (TIF 1.63 mb)

Supplementary Fig. 5 Comparative transcriptomic analysis of iNSCs and published transcriptomic datasets suggests a transcriptomic similarity of the converted NSCs with in vivo embryonic neuroepithelial cells. Pie chart exhibiting the fraction (%) of cells that belong to each cell type age based on the Mabbott et al. (**A**) and Eze et al. (**B**) SingleR age annotation

High resolution image (TIF 750 kb)

## Data Availability

The datasets used and/or analyzed during the current study are available from the corresponding author on reasonable request.

## Code Availability

The code used for the analysis during the current study will be available from the corresponding author on reasonable request.

## Abbreviations

*A*: Anterior
*hF*: Human fibroblast
*CNS*: Central nervous system
*D.W*.: Developmental week
*DEG*: Differentially expressed gene
*FMB *: Forebrain/ midbrain boundary
*iN*: Induced neuron
*iNSC*: Induced neural stem cell
*iPSC*: Induced pluripotent stem cell
*MHB*: Midbrain/ hindbrain boundary
*NIM*: Neural induction medium
*NSC*: Neural stem cell
*P*: Posterior
*R*: Rhombomere
*S.D*.: Spontaneously differentiating cells
*SeV*: Sendai virus
