# Supplementary figures and images for: Single-cell Profiling of Reprogrammed Human Neural Stem Cells Unveils High Similarity to Neural Progenitors in the Developing Central Nervous System
Source: Stem Cell Rev Rep. 2024 Mar 22;20(5):1325–39. doi: 10.1007/s12015-024-10698-3 (PMC11222274; doi:10.1007/s12015-024-10698-3)

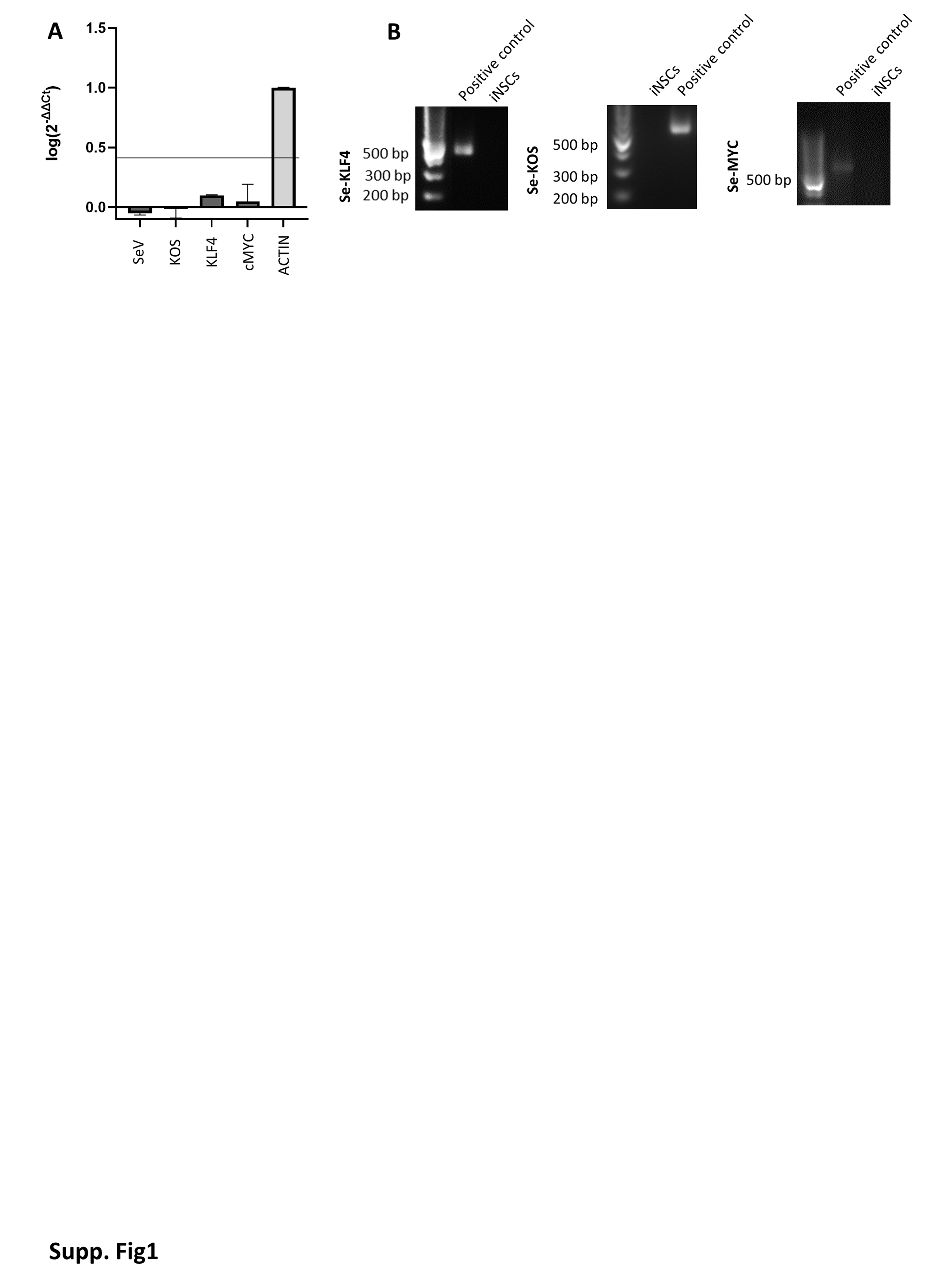

Supplement: Supplementary file 1 — Supplementary file1 Supplementary Fig. 1 The derived iNSCs do not maintain Sendai-OSKM expression. A. qPCR data showing that the iNSCs do not maintain any expression of the Sendai-associated OSKM genes. Error bars represent the mean ±S.E.M., n =3 technical replicates per gene. The horizontal line represents the expression threshold. B. Agarose gels showing no expression of the Sendai-associated OSKM genes. Clones that express OSKM were used as positive controls. [file 12015_2024_10698_Fig5_ESM.png]

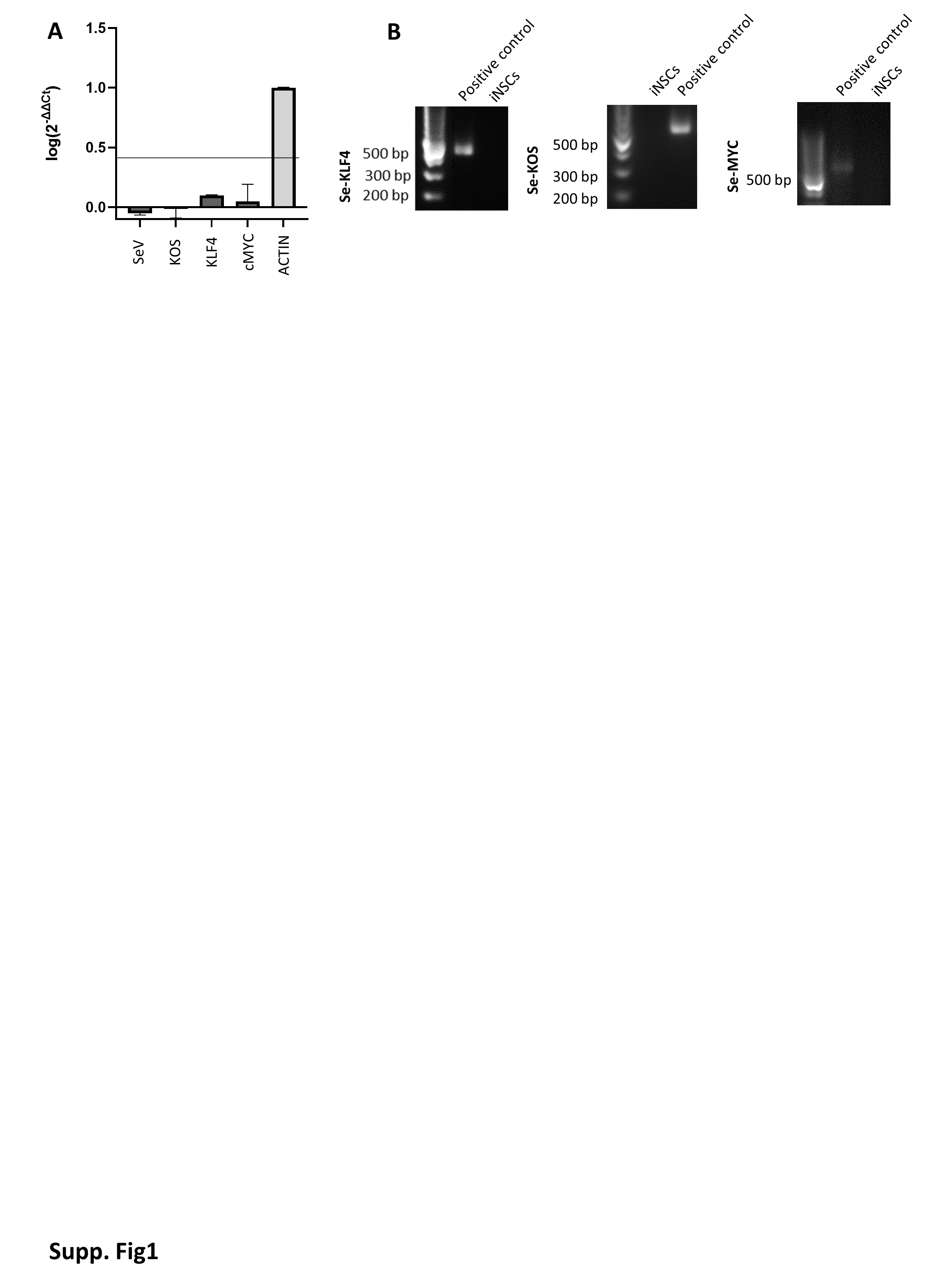

Supplement: Supplementary file 2 — High resolution image (TIF 764 kb) [file 12015_2024_10698_MOESM1_ESM.tif]

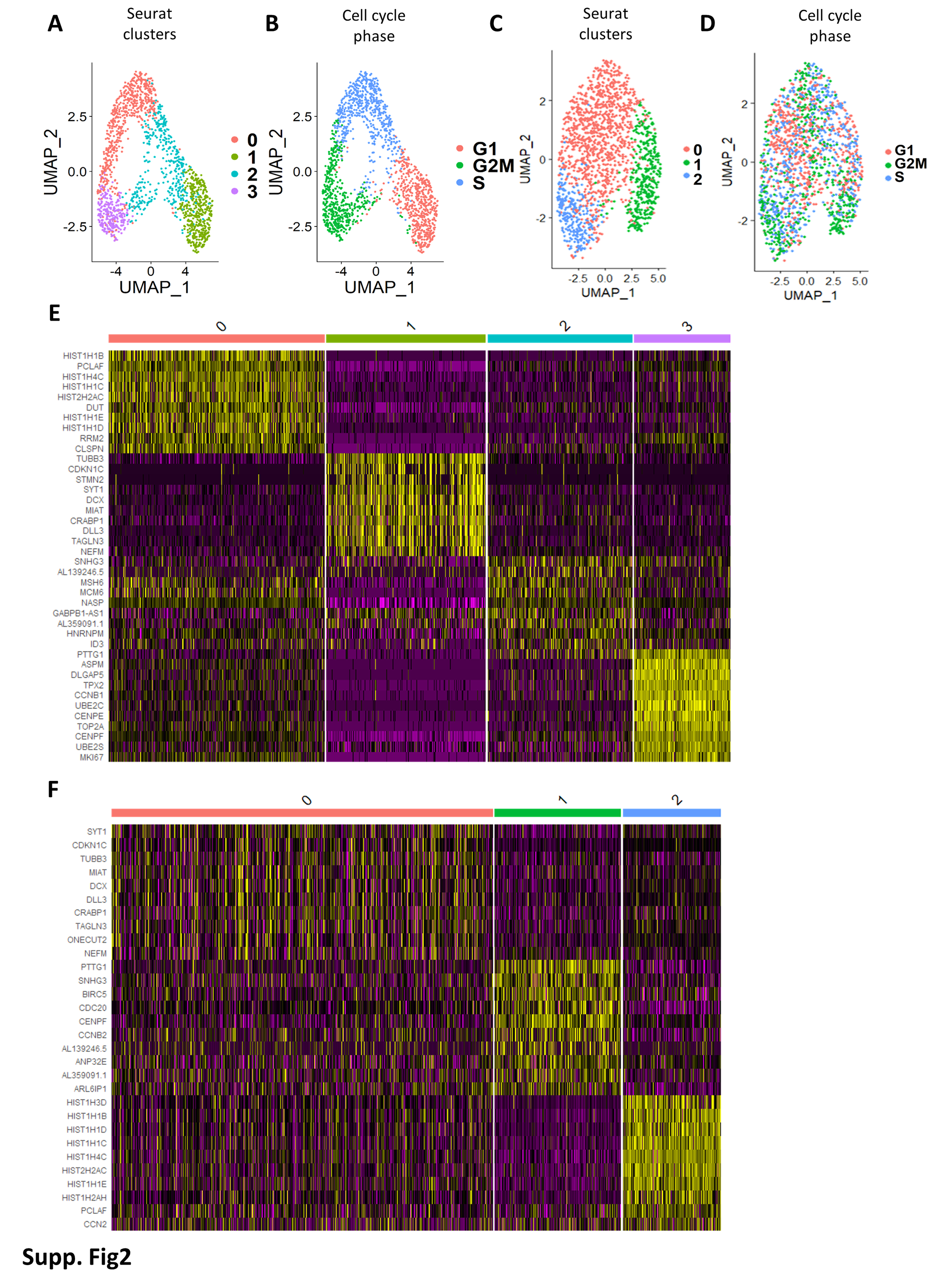

Supplement: Supplementary file 3 — Supplementary Fig. 2 UMAPs and heatmaps of the top 10 DEGs per cluster of the iNSC transcriptomic data. UMAP plots generated from iNSC scRNA-seq data clustered based on Seurat clustering (A) or their respective cell cycle phase (B). UMAP plots generated from iNSC scRNA-seq data after gene cell cycle regression. Clustering based on Seurat (C) or cell cycle phases (D), indicating a transcriptomically homogeneous NSC population after the cell cycle regression. Heatmap depicting the top 10 DEGs per cluster before (E) and after cell cycle gene regression (F). [file 12015_2024_10698_Fig6_ESM.png]

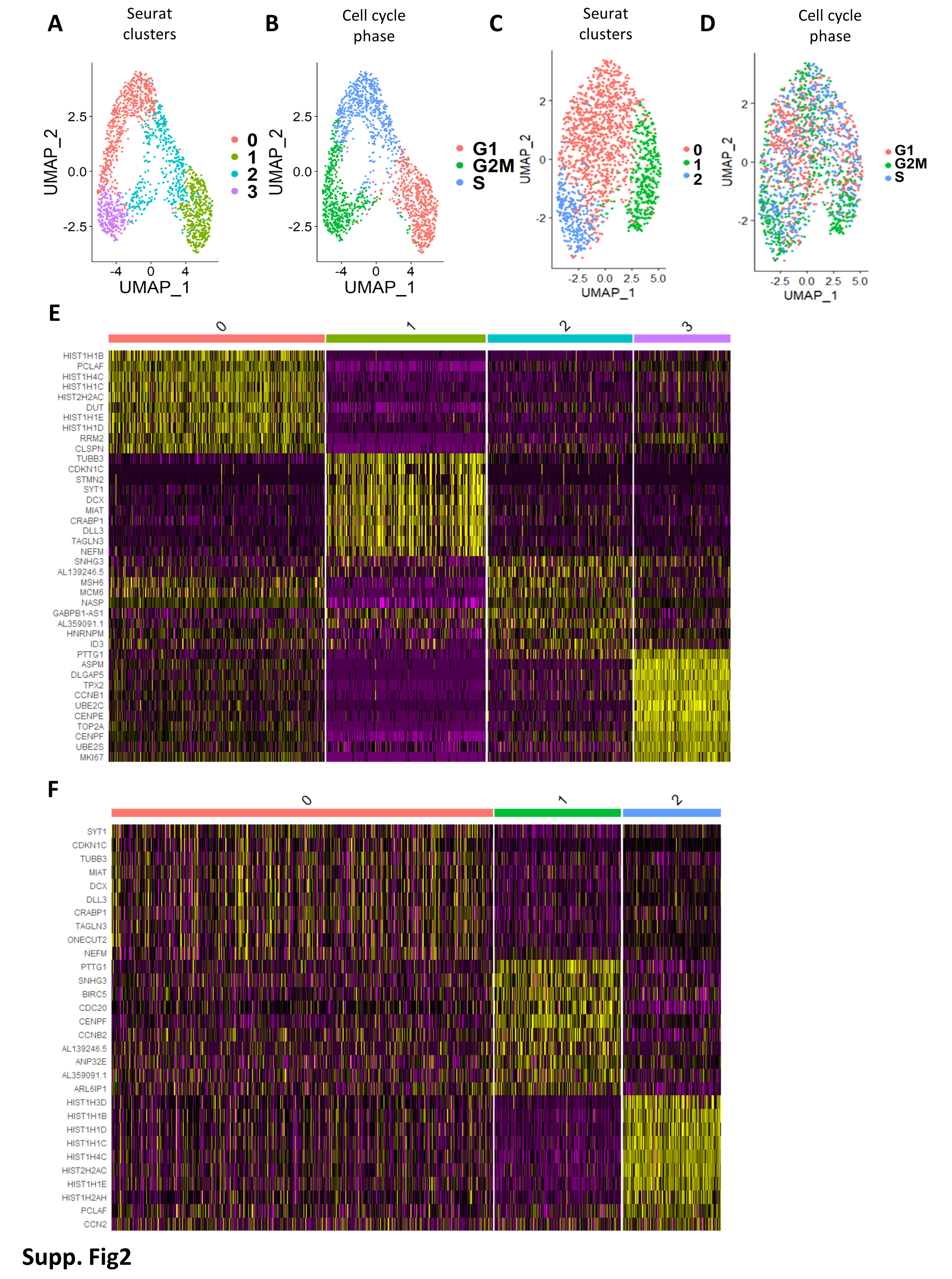

Supplement: Supplementary file 4 — High resolution image (TIF 10.8 mb) [file 12015_2024_10698_MOESM2_ESM.tif]

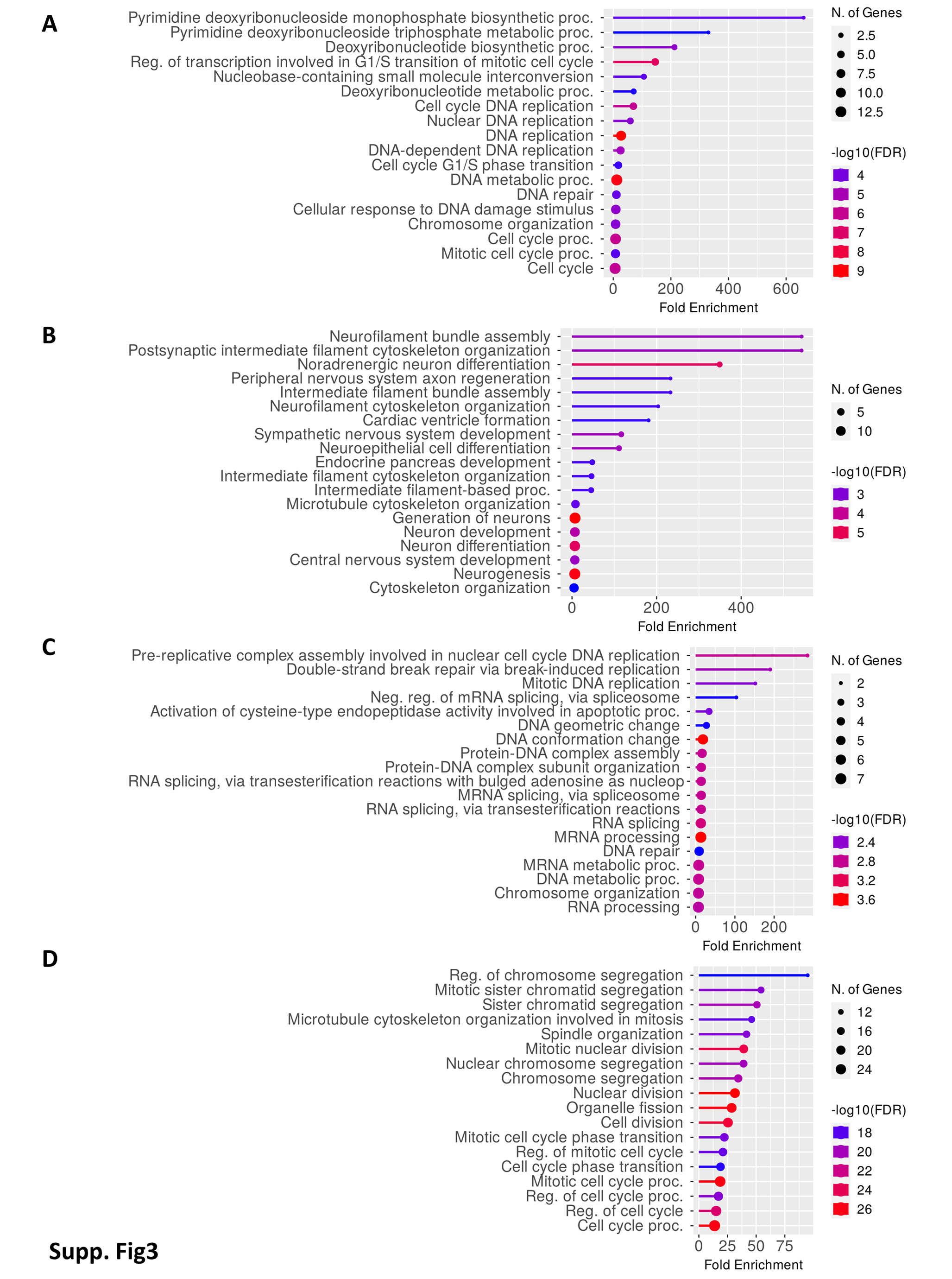

Supplement: Supplementary file 5 — Supplementary Fig. 3 GO terms per cluster of the iNSC transcriptomic data. Plots depicting the GO terms/ biological processes from cluster 0 (A), 1 (B), 2 (C) and 3 (D) as depicted in the UMAP plot of Supp. Fig. 2A. All plots are generated from the non-regressed scRNA-seq data. The plots are generated with the online bioinformatic tool ShinyGO 0.77. [file 12015_2024_10698_Fig7_ESM.png]

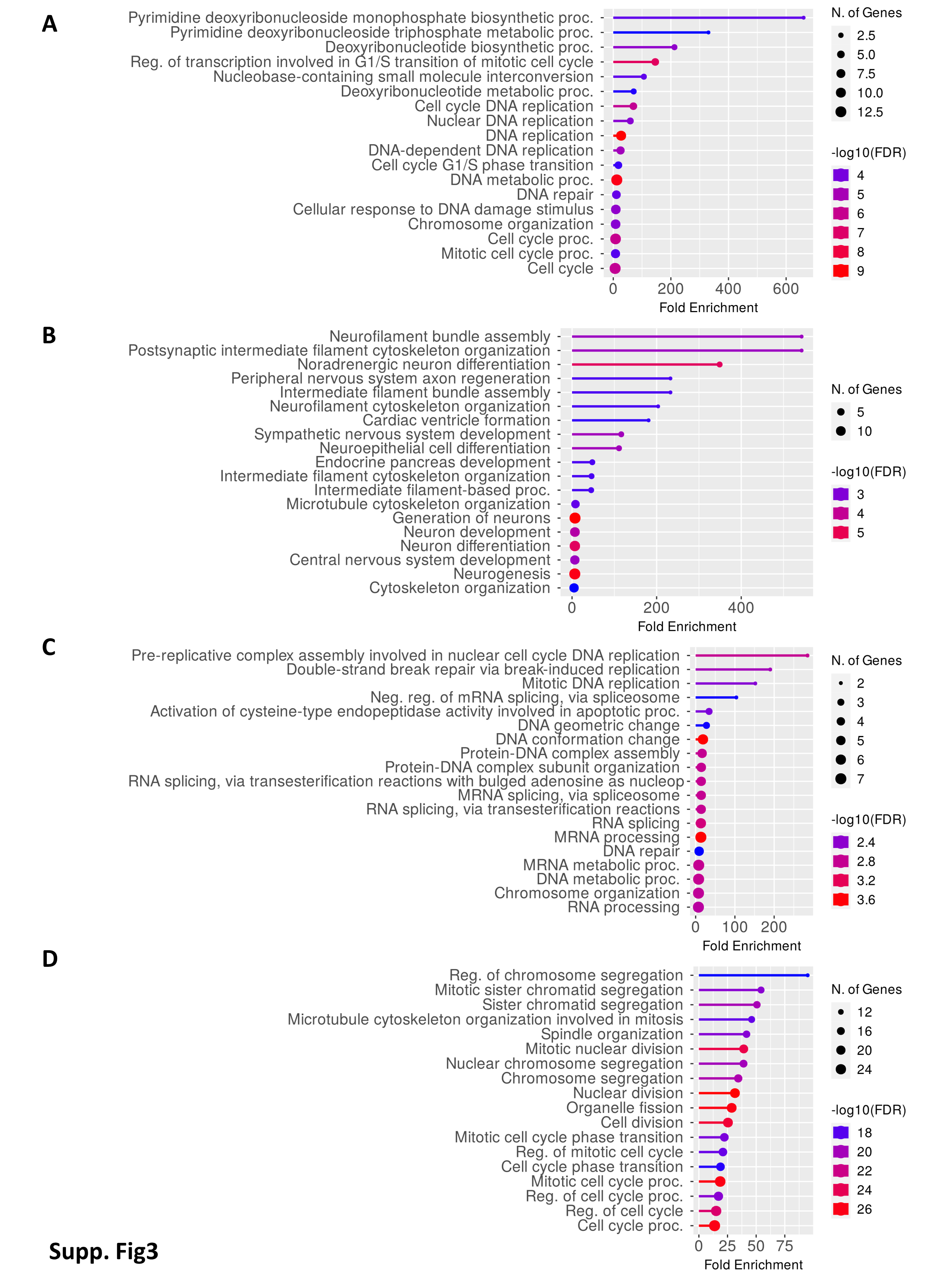

Supplement: Supplementary file 6 — High resolution image (TIF 2.06 mb) [file 12015_2024_10698_MOESM3_ESM.tif]

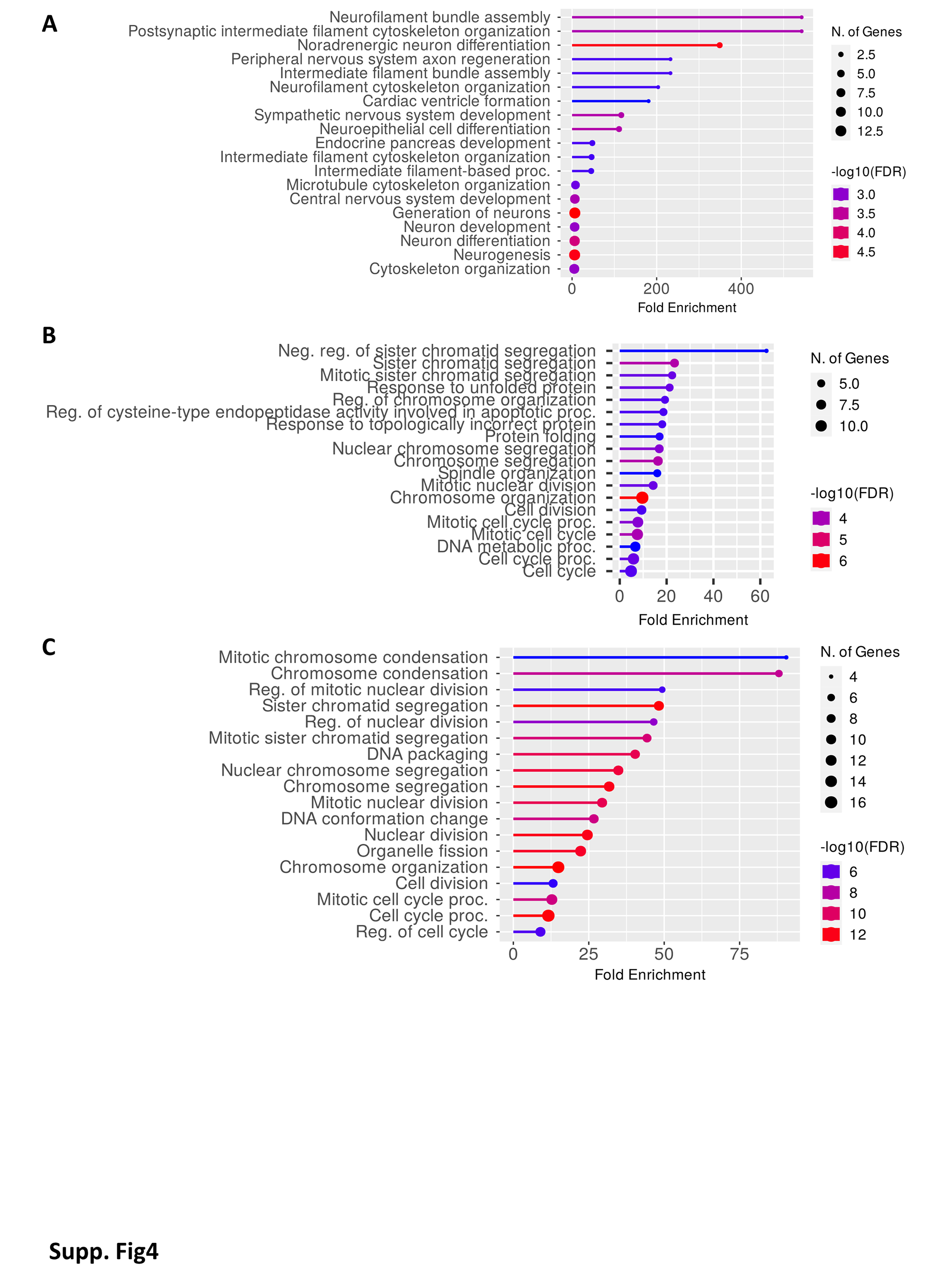

Supplement: Supplementary file 7 — Supplementary Fig. 4 GO terms per cluster of the cell cycle-regressed iNSC transcriptomic data. Plots depicting the GO terms/ biological processes from cluster 0 (A), 1 (B), and 2 (C) as depicted in the UMAP plot of Supp. Fig. 2C. All plots are generated from the cell cycle-regressed scRNA-seq data. The plots are generated with the online bioinformatic tool ShinyGO 0.77. [file 12015_2024_10698_Fig8_ESM.png]

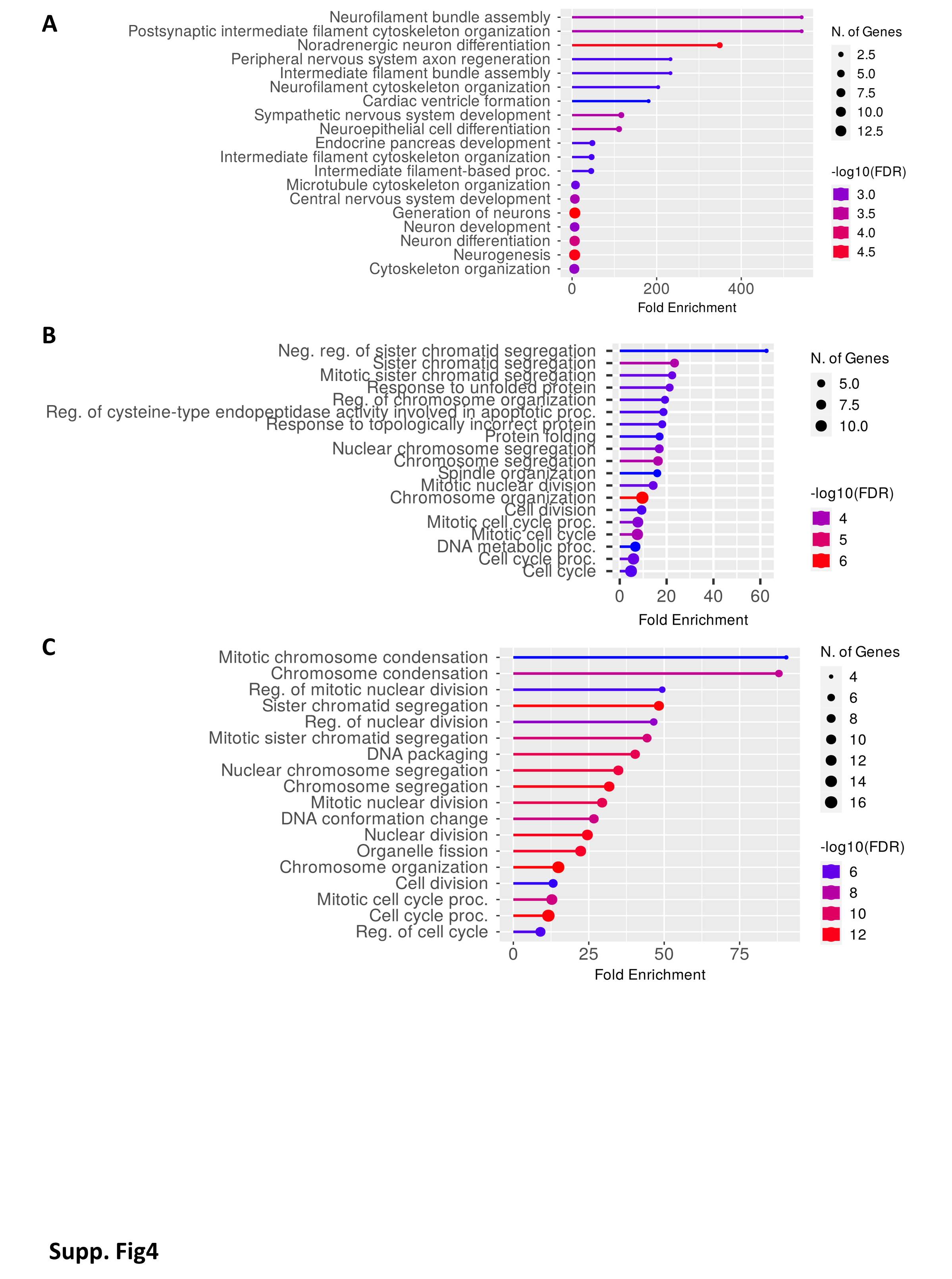

Supplement: Supplementary file 8 — High resolution image (TIF 1.63 mb) [file 12015_2024_10698_MOESM4_ESM.tif]

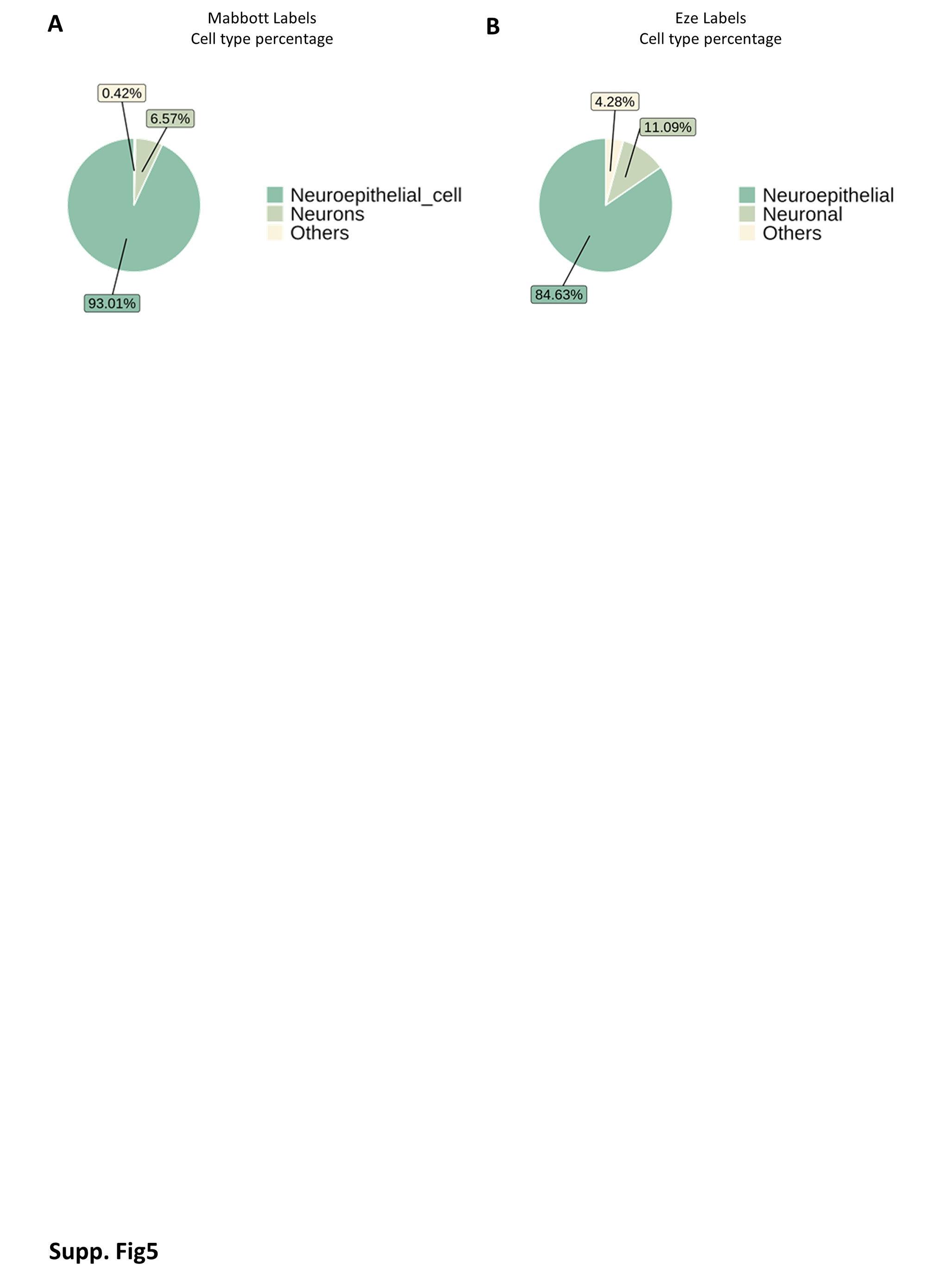

Supplement: Supplementary file 9 — Supplementary Fig. 5 Comparative transcriptomic analysis of iNSCs and published transcriptomic datasets suggests a transcriptomic similarity of the converted NSCs with in vivo embryonic neuroepithelial cells. Pie chart exhibiting the fraction (%) of cells that belong to each cell type age based on the Mabbott et al. (A) and Eze et al. (B) SingleR age annotation [file 12015_2024_10698_Fig9_ESM.png]

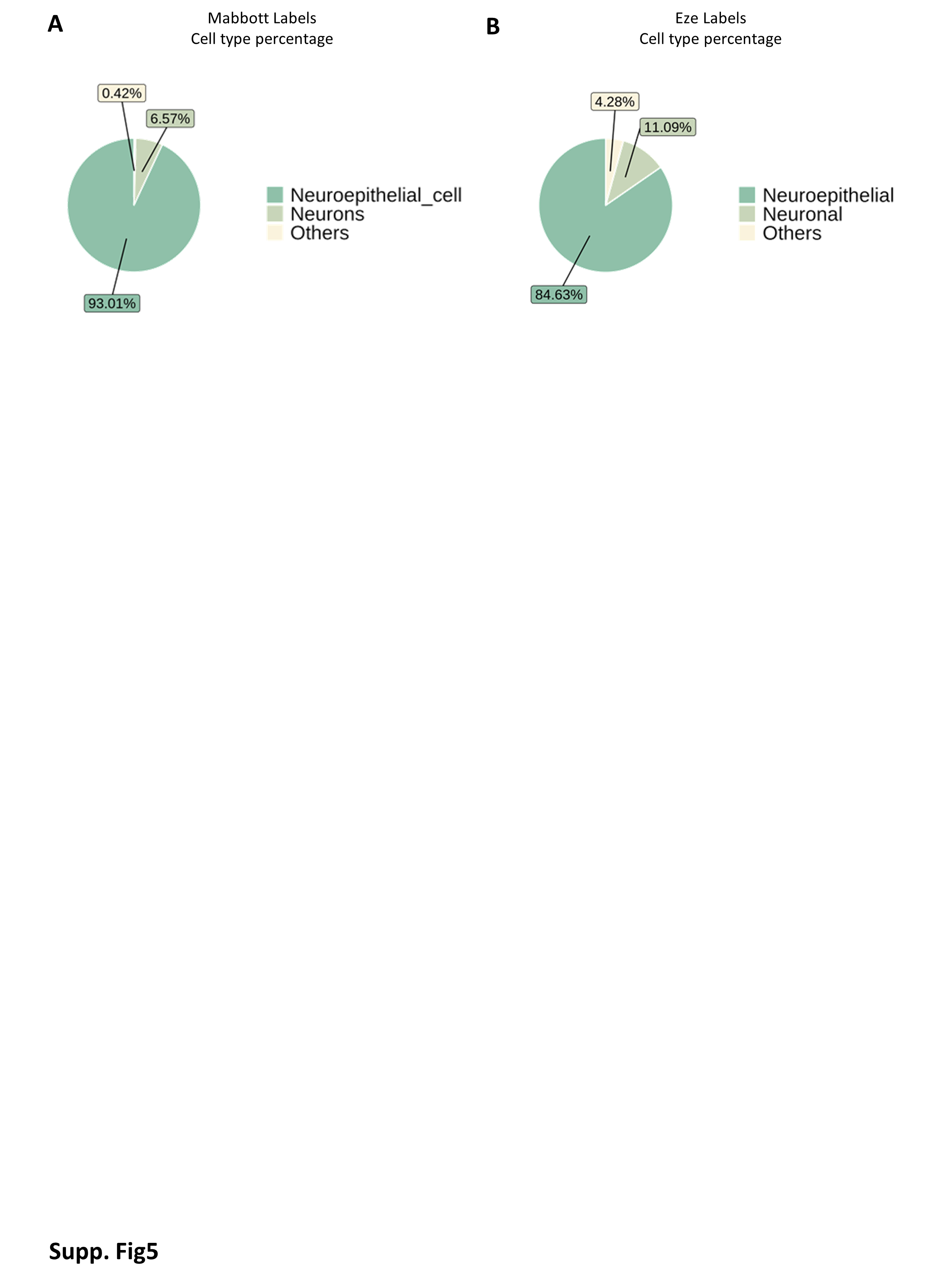

Supplement: Supplementary file 10 — High resolution image (TIF 750 kb) [file 12015_2024_10698_MOESM5_ESM.tif]
